# Individualized dynamic frailty-tailored therapy (DynaFiT) in elderly patients with newly diagnosed multiple myeloma: a prospective study

**DOI:** 10.1186/s13045-024-01569-y

**Published:** 2024-06-24

**Authors:** Yingjie Zhang, Xinyue Liang, Weiling Xu, Xingcheng Yi, Rui Hu, Xintian Ma, Yurong Yan, Nan Zhang, Jingxuan Wang, Xiaoxiao Sun, Yufeng Zhu, Mengru Tian, Maozhuo Lan, Mengtuan Long, Yun Dai, Fengyan Jin

**Affiliations:** 1 Department of Hematology, First Hospital of Jilin University, 71 Xinmin Street, Changchun, Jilin 130012 China; 2 Laboratory of Cancer Precision Medicine, First Hospital of Jilin University, 519 Dongminzhu Street, Changchun, Jilin 130061 China; 3 Department of Radiology, First Hospital of Jilin University, Changchun, Jilin China

**Keywords:** Frailty, Dynamics, Frailty-tailored therapy, Elderly, Multiple myeloma

## Abstract

It remains a substantial challenge to balance treatment efficacy and toxicity in geriatric patients with multiple myeloma (MM), primarily due to the dynamic nature of frailty. Here, we conducted a prospective study to evaluate the feasibility and benefits of dynamic frailty-tailored therapy (DynaFiT) in elderly patients. Patients with newly diagnosed MM (aged ≥ 65 years) received eight induction cycles of bortezomib, lenalidomide, and dexamethasone (daratumumab was recommended for frail patients), with treatment intensity adjusted according to longitudinal changes in the frailty category (IMWG-FI) at each cycle. Of 90 patients, 33 (37%), 16 (18%), and 41 (45%) were fit, intermediate fit, and frail at baseline, respectively. Of 75 patients who had geriatric assessment at least twice, 28 (37%) experienced frailty category changes at least once. At analysis, 15/26 (58%) frail patients improved (27% became fit and 31% became intermediate fit), 4/15 (27%) intermediate fit patients either improved or deteriorated (two for each), and 6/30 (20%) fit patients deteriorated. During induction, 34/90 (38%) patients discontinued treatment, including 10/33 (30%) fit, 4/16 (25%) intermediate fit, and 20/41 (49%) frail; 14/40 (35%) frail patients discontinued treatment within the first two cycles, mainly because of non-hematologic toxicity (mostly infections). For fit, intermediate-fit, and frail patients, the overall response rate was 100%, 93%, and 73%, respectively; one-year overall survival was 90%, 75%, and 54%, respectively. Therefore, the individualized DynaFiT is feasible and promising for heterogeneous elderly patients.

**Supplementary Information:**

The online version contains supplementary material available at 10.1186/s13045-024-01569-y.

To the editor

Despite a remarkable improvement in the outcome of patients with multiple myeloma (MM), the benefit is considerably less impressive for elderly patients [1, 2], mainly because of treatment discontinuation (TD) due to frailty [3–6]. While frailty shows dynamic [7, 8], it is challenging to treat elderly patients featured by longitudinal frailty changes. To this end, we conducted a prospective study to investigate the feasibility and benefits of an individualized dynamic frailty-tailored therapy (DynaFiT) in elderly patients with newly diagnosed MM (NDMM).

This study was designed based on real-life practice at our center, which enrolled patients aged ≥ 65 years with NDMM who were transplant-ineligible or had no intent for immediate transplant, with minimal exclusion criteria (see Supplementary Information for Methods in detail). According to the NCCN Guidelines Insights: Multiple Myeloma (version 1.2020), participants received eight 21-day cycles of bortezomib, lenalidomide, and dexamethasone (VRd) for induction, followed by maintenance with Rd. Based on the EMN recommendation [9], treatment intensity was adjusted according to longitudinal changes in the frailty category, defined by the IMWG-FI [10], at the start of each cycle (Fig. S1). Daratumumab was recommended for frail patients [3–5]. Antibiotic/antiviral prophylaxis was recommended according to the IMWG’s consensus [11].

From August 2021 to September 2023, 105 patients were registered, of whom 15 were deemed ineligible (Fig. S2). The baseline characteristics of 90 eligible patients are summarized in Table S1, of whom 33 (37%), 16 (18%), and 41 (45%) were fit, intermediate fit, and frail (Table S2), and their baseline characteristics are compared in Table S3.

At analysis, 75 patients had frailty assessment at least twice, of whom 28 (37%) experienced a change in the frailty category at least once during induction (Table 1). Of 41 frail patients (Fig. 1a), 11 patients had only baseline frailty assessment and four were age > 80 years; of 26 analyzable patients, 15 (58%) became fit (27%) or intermediate fit (31%), because of increased IADL, ADL, or both scores; of 15 patients (including two aged ≥ 80 years) receiving daratumumab, eight (62%) had an improvement. Of 30 fit patients (Fig. 1b), six (20%) became intermediate fit or frail, due to reduced IADL, ADL and IADL (because of grade 2 peripheral neuropathy with pain), or ADL plus age turning > 75 years. Of 15 intermediate-fit patients (Fig. 1c), two (13%) became fit due to increased ADL or IADL, and two became frail due to reduced IADL or ADL and IADL. 36/90 (40%) patients proceeded to maintenance, with trajectories of the frailty category from baseline to maintenance initiation shown in Fig. 1d. Of 34 patients with ECOG 3 or 4, 11 (32%) had an improvement in the frailty score, and 7 (21%) were tolerated to protocol treatment (though no improvement in the frailty score), while 16 (47%) discontinued treatment due to AEs (10), deteriorating conditions (3), noncompliances (2), or COVID-19 (1).

**Table 1:** Frailty category changes, therapeutic responses, and treatment discontinuation

	All, N (%)	Fit, N (%)	Intermediate fit, N (%)	Frail, N (%)
**Frailty category change**	**(N = 75)**	**(N = 30)**	**(N = 15)**	**(N = 30)**
At least once	28 (37.3)	8 (26.7)	5 (33.3)	15 (50.0)^a^
At analysis				
Improved	17 (22.7)	−	2 (13.3)	15 (50.0)
Deteriorated	8 (10.7)	6 (20.0)	2 (13.3)	−
**Response**	**(N = 74)**	**(N = 30)**	**(N = 14)**	**(N = 30)**
ORR	65 (87.8)	30 (100)	13 (92.9)	22 (73.3)
CR or sCR	40 (54.1)	18 (60.0)	9 (64.3)	13 (43.3)
VGPR	15 (20.3)	8 (26.7)	3 (21.4)	4 (13.3)
PR	10 (13.5)	4 (13.3)	1 (7.1)	5 (16.7)
MR	4 (5.4)	0 (0)	1 (7.1)	3 (10.0)
SD	5 (6.8)	0 (0)	0 (0)	5 (16.7)
**Reason for TD**	**(N = 90)**	**(N = 33)**	**(N = 16)**	**(N = 41)**
Non-hematologic AE, any	17 (18.9)	5 (15.2)	1 (6.3)	11 (26.8)
Grade 2				
PNP-P	2 (2.2)	2 (6.1)	−	−
Grade 3				
Pneumonia	1 (1.1)	−	−	1 (2.4)
Cerebral infarction	1 (1.1)	−	−	1 (2.4)
Grade 4				
Pneumonia	5 (5.6)	1 (3.0)	−	4 (9.8)
Sepsis	4 (4.4)	1 (3.0)	−	3 (7.3)
Acute heart failure	1 (1.1)	1 (3.0)	−	−
Intercurrent death				
Sudden death	2 (2.2)	−	1 (6.3)	1 (2.4)
Acute renal failure	1 (1.1)	−	−	1 (2.4)
Hematologic AE				
Thrombocytopenia	1 (1.1)	−	−	1 (2.4)^b^
Reason other than AE, any	16 (17.8)	5 (15.2)	3 (18.8)	8 (19.5)
Deteriorating condition	4 (4.4)	−	1 (6.3)	3 (7.3)
Disease progression	3 (3.3)	1 (3.0)	1 (6.3)	1 (2.4)
Noncompliance	6 (6.7)	2 (6.1)	1 (6.3)	3 (7.3)
COVID-19	3 (3.3)	2 (6.1)	−	1 (2.4)

Abbreviations: ORR, overall response rate; sCR, stringent complete response; CR, complete response; VGPR, very good partial response; PR, partial response; MR, minimal response; SD, stable disease; PFS, progression-free survival; OS, overall response; TD, treatment discontinuation; AE, adverse event; PNP-P, peripheral neuropathy grade 2 with pain.

^a^Including four patients with age > 80 years.

^b^TD because of cerebral hemorrhage secondary to thrombocytopenia.

**Fig. 1:**
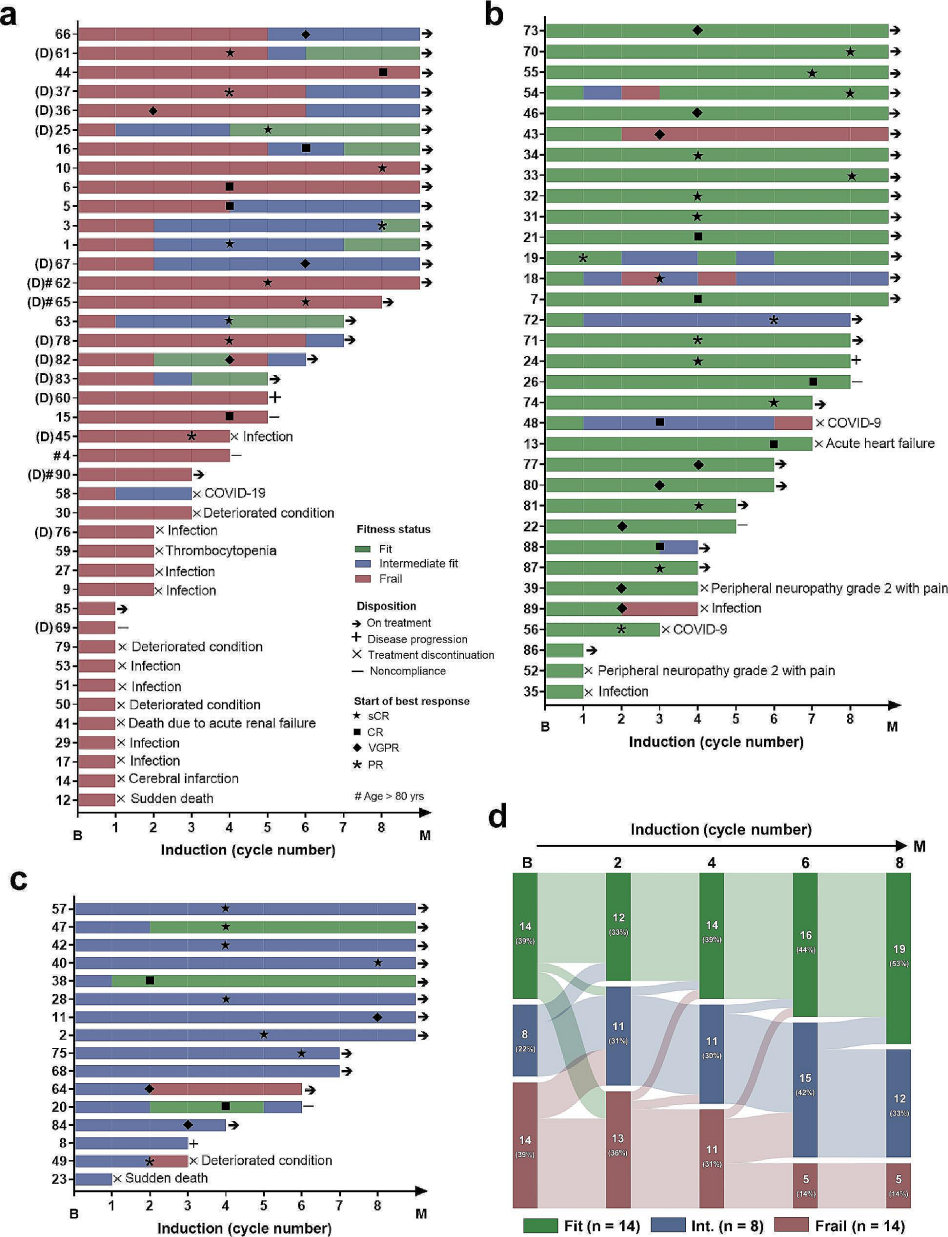
Longitudinal changes of frailty. Changes in the frailty category during induction for patients who were defined as frail (**a**; patient #4 based on age > 80 years alone), fit **(b)**, and intermediate fit **(c)** at baseline, as well as who proceeded to maintenance **(d)**. B, baseline; M, maintenance; D, daratumumab; sCR, stringent complete response; CR, complete response; VGPR, very good partial response; PR, partial response

Of 74 patients evaluable for responses, the ORR was 88% (Table 1), including (s)CR (54%), VGPR (20%), and PR (14%). The ORR was 100%, 93%, and 73% for fit, intermediate-fit, and frail patients. Only one patient experienced progression during induction in each group. One-year PFS and OS were 85% and 90% for fit, 75% each for intermediate fit, and 46% and 54% for frail, respectively.

34/90 (38%) discontinued the protocol treatment, with 10/33 (30%), 4/16 (25%), and 20/41 (49%) for fit, intermediate fit, and frail, respectively (Table S4). The reasons for TD are described in Table 1, including five non-hematologic AEs, two noncompliances, two COVID-19s, and one PD for fit; intercurrent death, deteriorating condition, noncompliance, and PD (one for each) for intermediate fit; eleven non-hematologic AEs, three deteriorating conditions, three noncompliances, one cerebral hemorrhage secondary to thrombocytopenia, one PD, and one COVID-19 for frail. Of note, TD due to toxicity (mostly infections) accounted for 93% of frail patients who discontinued treatment within the first two cycles. Of 15 frail patients receiving additional daratumumab, only two (13%) discontinued treatment due to AEs.

Cumulative grade ≥ 3 non-hematologic and hematologic toxicities were reported in 43 (48%) and 45 (50%) of 90 patients (Table S5). Cumulative grade ≥ 3 non-hematologic toxicities were reported in 45% (15/33), 50% (8/16), and 49% (20/41) of fit, intermediate-fit, and frail patients, respectively (Table S6). Three patients (one intermediate fit and two frail) died during the first cycle (early mortality, 3.3%), with two sudden unexplained deaths and one due to acute renal failure.

In summary, we report for the first time, to our knowledge, that the DynaFiT is feasible for elderly patients in real-life practice. It allows timely adjusting of treatment intensity to balance efficacy and safety during treatment according to longitudinal changes in the frailty category to avoid both undertreatment and overtreatment in this heterogeneous population. While the choice of treatment in older patients based on the decision of the physician could be safe and effective [12], the DynaFiT may change the view of managing frail patients, to whom intensive therapy was generally not recommended [10]. Moreover, this study may also serve as a prototype for future studies to investigate other regimens in elderly patients with MM.

### Electronic supplementary material

Below is the link to the electronic supplementary material.

Supplementary Material 1

## Data Availability

No datasets were generated or analysed during the current study.
