# Supplementary material for: Individualized dynamic frailty-tailored therapy (DynaFiT) in elderly patients with newly diagnosed multiple myeloma: a prospective study
Source: J Hematol Oncol. 2024 Jun 24;17:48. doi: 10.1186/s13045-024-01569-y (PMC11197371; doi:10.1186/s13045-024-01569-y)
Supplement: Supplementary file 1 — Supplementary Material 1 [file 13045_2024_1569_MOESM1_ESM.doc]

**Individualized dynamic frailty-tailored therapy (DynaFiT) in elderly patients with newly diagnosed multiple myeloma: a prospective study**

**Methods**

**Study design and treatment**

This study was designed based on our real-life practice. According to the NCCN Guidelines Insights: Multiple Myeloma (version 1.2020) for transplant-ineligible patients with NDMM, participants received eight cycles of bortezomib, lenalidomide, and dexamethasone (VRd) for induction therapy, followed by maintenance with Rd until progression or intolerance. Because elderly patients with NDMM seen at our center often had advanced diseases (ISS III) and organ involvement, including renal impairment (eGFR <60 ml/min/1.73m^2^) and lytic bone lesions [1], 21-day cycle was chosen due to the high prevalence of advanced disease in this study. Based on the European Myeloma Network (EMN) recommendation and the findings of previous studies [2-5], treatment intensity was adjusted according to longitudinal changes in the frailty category (according to the IMWG-FI) at the start of each cycle: fit, standard-dose VRd (level 0) consisting of V (1.3 mg/m^2^; days 1, 4, 8, and 11), R (25 mg/day; for 14 days), and d (20 mg; days 1, 4, 8, and 11); intermediate fit, dose-reduced VRd (level -1) consisting of V (1.3 mg/m^2^; days 1, 4, 8, and 11), R (15 mg/day; for 14 days), and d (10 mg; days 1, 4, 8, and 11); frail, dose-reduced Vd (level -2) consisting of V (1.0 mg/m^2^; day 1, 4, 8, and 11) and d (5 mg; days 1, 4, 8, and 11). For patients who had renal impairment, lenalidomide dosing was adjusted by following the recommendations in the NCCN Guidelines Insights: Multiple Myeloma (version 1.2020), regardless of their frailty status. Addition of daratumumab (16 mg/kg; cycle 1-2, days 1, 8, 15, and 22; cycle 3-6, days 1 and 15; cycle 7-8, day 1) for induction, followed by maintenance with daratumumab (day 1) until progression or a maximum of two years [6], was recommended for frail patients. The dose and schedule of daratumumab were not adjusted or discontinued when frail patients became fit or intermediate fit. Since the IMWG's consensus for infection prevention in MM was issued on February 2022 [7], antibiotic (e.g., levofloxacin) and antiviral prophylaxis (e.g., acyclovir) was recommended.

**IMWG frailty index**

The frailty/fitness statuses were categorized by the International Myeloma Working Group frailty index (IMWG-FI) [8], based on age, comorbidities, dependency in activities of daily living (ADL) and instrumental ADL (IADL). The total score ranges from 0-5 points. Patients are defined as fit (0 point), intermediate fit (1 point), and frail (≥ 2 points).

| **Parameter** | **Points** | |
| --- | --- | --- |
| **Age, years** |  | |
| ≤ 75 | | 0 |
| 76-80 | | 1 |
| > 80 | | 2 |
| **Charlson comorbidity index (CCI)** [9] | |  |
| ≤ 1 | | 0 |
| ≥ 2 | | 1 |
| **Activity of daily living (ADL)** [10] | |  |
| > 4 | | 0 |
| ≤ 4 | | 1 |
| **Instrumental activity of daily living (IADL)** [11] | |  |
| > 5 | | 0 |
| ≤ 5 | | 1 |
| **IMWG-FI category** | |  |
| Fit | | 0 |
| Intermediate fit | | 1 |
| Frail | | ≥ 2 |

**Inclusion criteria**

- Age ≥ 65 years, either male or female;
- Previous untreated patients with a confirmed diagnosis with multiple myeloma (MM) according to the IMWG criteria [12];
- Written informed consent (with the understanding that consent may be withdrawn by the patient at any time without consequences to future medical care).

**Exclusion criteria**

- Plasma cell leukemia;
- Systemic amyloid light-chain amyloidosis;
- Neuropathy, grade 1 with pain or grade ≥ 2;
- Severe cardiac dysfunction (NYHA classification III-IV, unstable angina pectoris, grade IV congestive heart failure, or uncontrolled malignant arrhythmia), except those caused by cardiac amyloidosis secondary to MM;
- Severe hepatic dysfunction (total bilirubin ≥ 3 x ULN or transaminases ≥ 5 normal level), except those caused by hepatic amyloidosis secondary to MM;
- Patients with active, uncontrolled infections;
- Active malignancy other than MM requiring treatment or a malignancy that has been treated with chemotherapy within 5 years.

**Outcomes**

The primary endpoint of this study was overall survival (OS, defined as the time from enrollment to death due to any causes). Other key endpoints of this analysis (Supplement Methods) included TD (defined as cessation of the protocol treatment during induction), adverse events (AEs), overall response rate (ORR, defined as a partial response/PR or better), and one-year OS and progression-free survival (PFS, defined as the time from enrollment to progression or death due to any causes). Therapeutic responses were evaluated according to the International Response Criteria for Multiple Myeloma [13]. AEs were graded according to the National Cancer Institute Common Terminology Criteria for Adverse Events (CTCAE version 5.0) [14].

**Definition of responses and endpoints**

- - Objective response, defined as a partial response or better;
  - Best response, defined as a complete response or better;
  - Overall survival, defined as the time from enrollment to death due to any causes;
  - Progression-free survival, defined as the time from enrollment to progression or death due to any causes;
  - Early mortality, defined as death within 60 days from treatment initiation [6];
  - Treatment discontinuation, defined as cessation of the protocol treatment during induction;
  - In our pilot study, 40.8% of participants discontinued treatment during eight cycles of induction with the DynaFiT, lower than those reported for frail (51%) and intermediate-fit patients (46%) in the Hovon 143 study [6,15]. Accordingly, this therapy is considered feasible if less than 40% of the participants discontinued the protocol treatment due to any reason during eight cycles of induction.

**Statistical analysis**

Simon's two-stage design v1.0.2.0 (https://biostatistics.mdanderson.org/shinyapps/ Simon2S/) was used to calculate sample size, with α (one-sided) of 0.05 and a power (1−β) of 80%. With an unpromising ORR (p0) of 50% and desirable ORR (p1) of 65% [6], the optimal sample size was 83. Considering an ineligible rate of 25% (according to our pilot study), 104 patients were needed. Observations were censored on November 30, 2023. EFS and time to TD (TTD) were analyzed using the Kaplan-Meier method and log-rank test (two-sided). Hazard ratios (HRs) with 95% CI were estimated by the Cox proportional hazards model. Statistical analyses were performed using SPSS software (version 26.0) and R packages survival and survminer in R/Bioconductor (version 4.0.4). *P* < 0.05 was considered statistically significant.

**Limitations**

First, 21-day cycle (bortezomib twice weekly), rather than 28-day cycle (bortezomib once weekly) recommended by the EMN [4], was chosen due to the high prevalence of advanced disease in the participants of this study. However, bortezomib twice weekly is more toxic, which might contribute to a high incidence of AEs (e.g., infections) and their related TD in frail patients. Second, although it was recommended to frail patients, only 37% of them received daratumumab because this study was initiated before this novel agent was covered by health insurance in China until the end of 2023. Considering its favorable safety profile for elderly patients [15], it would be expected that the incorporation of daratumumab into the DynaFiT might help reduce the TD rate in frail patients. Last, only 31% of participants received antibiotic and antiviral prophylaxis because this study was initiated before the IMWG's consensus for infection prevention in MM was issued in February 2022 [7]. This might also represent one of the reasons for the high incidence of infections, particularly in frail patients.

**References**

1. Zhang Y, Xue H, Li M, Xu JM, Liang XY, Xu WL, et al. A multicenter study of R-ISS staging combined with frailty biomarkers to predict the prognosis and early death in newly diagnosed elderly multiple myeloma patients. Chin J Geriatr. 2023;42:1207-12. doi: 10.3760/cma.j.issn.0254-9026.2023.10.011.
2. Facon T, Leleu X, Manier S. How I treat multiple myeloma in geriatric patients. Blood. 2024;143:224-32. doi: 10.1182/blood.2022017635.
3. Lee HC, Ailawadhi S, Gasparetto CJ, Jagannath S, Rifkin RM, Durie BGM, et al. Treatment patterns and outcomes in elderly patients with newly diagnosed multiple myeloma: results from the Connect® MM Registry. Blood Cancer J. 2021;11:134. doi: 10.1038/s41408-021-00524-1.
4. Larocca A, Dold SM, Zweegman S, Terpos E, Wäsch R, D'Agostino M, et al. Patient-centered practice in elderly myeloma patients: an overview and consensus from the European Myeloma Network (EMN). Leukemia. 2018;32:1697-712. doi: 10.1038/s41375-018-0142-9.
5. O'Donnell EK, Laubach JP, Yee AJ, Chen T, Huff CA, Basile FG, et al. A phase 2 study of modified lenalidomide, bortezomib and dexamethasone in transplant-ineligible multiple myeloma. Br J Haematol. 2018;182:222-30. doi: 10.1111/bjh.15261.
6. Stege CAM, Nasserinejad K, van der Spek E, Bilgin YM, Kentos A, Sohne M, et al. Ixazomib, daratumumab, and low-dose dexamethasone in frail patients with newly diagnosed multiple myeloma: The Hovon 143 study. J Clin Oncol. 2021;39:2758-67. doi: 10.1200/JCO.20.03143.
7. Raje NS, Anaissie E, Kumar SK, Lonial S, Martin T, Gertz MA, et al. Consensus guidelines and recommendations for infection prevention in multiple myeloma: a report from the International Myeloma Working Group. Lancet Haematol. 2022;9:e143–61. doi: 10.1016/S2352-3026(21)00283-0.
8. Palumbo A, Bringhen S, Mateos M-V, Larocca A, Facon T, Kumar SK, et al. Geriatric assessment predicts survival and toxicities in elderly myeloma patients: an International Myeloma Working Group report. Blood 125:2068-2074, 2015.
9. Charlson ME, Pompei P, Ales KL, Mackenzie CR. A new method for classifying prognostic comorbidity in longitudinal studies: development and validation. J Chron Dis 40:373-383, 1987.
10. Katz S, Ford AB, Moskowitz RW, Jackson BA, Jaffe MW. Studies of Illness in the Aged. The index of ADL: a standardized measure of biological and psychosocial function. JAMA. 185:914-919, 1963.
11. Lawton MP, Brody EM. Assessment of older people: self-maintaining and instrumental activities of daily living. Gerontologist 9:179-186, 1969.
12. Rajkumar SV, Dimopoulos MA, Palumbo A, Blade J, Merlini G, Mateos M-V, et al. International Myeloma Working Group updated criteria for the diagnosis of multiple myeloma. Lancet Oncol 15:e538-e548, 2014.
13. Kumar SK, Paiva B, Anderson KC, Durie B, Landgren O, Moreau P, et al. International Myeloma Working Group consensus criteria for response and minimal residual disease assessment in multiple myeloma. Lancet Oncol 17:e328–e346, 2016.
14. US Department of Health and Human Services, National Institutes of Health, National Cancer Institute. Common Terminology Criteria for Adverse Events (CTCAE) version 4.0. May 28, 2009.
15. Tyczyńska A, Zaucha J. At what point are we on the way to optimally treat multiple myeloma patients over 75 years of age in 2023? Adv Clin Exp Med 33:409-418, 2024.

**Supplementary Data**

**Table S1. Patient c**haracteristics

| **Characteristic** | | **N (%)** |
| --- | --- | --- |
| **Age, years** | | **(n = 90)** |
| Median (range) | 70 (65−87) | |
| 65-70 | 53 (58.9) | |
| 71-75 | 25 (27.8) | |
| 76-80 | 8 (8.9) | |
| > 80 | 4 (4.4) | |
| **Sex** | **(n = 90)** | |
| Female | 44 (48.9) | |
| Male | 46 (51.1) | |
| **M protein** | **(n = 90)** | |
| IgG | 38 (42.2) | |
| IgA | 19 (21.1) | |
| IgD | 6 (6.7) | |
| Light chain | 23 (25.6) | |
| Non/oligosecretory | 4 (4.4) | |
| **ISS** | **(n = 89)** | |
| I | 7 (7.9) | |
| II | 25 (28.1) | |
| III | 57 (64.0) | |
| **R-ISS** | **(n = 83)** | |
| I | 4 (4.8) | |
| II | 55 (66.3) | |
| III | 24 (28.9) | |
| **BMPC** | **(n = 90)** | |
| ≥ 30% | 53 (58.9) | |
| < 30% | 37 (41.1) | |
| **β2-MG** | **(n = 89)** | |
| ≥ 5.5 mg/L | 57 (64.0) | |
| < 5.5 mg/L | 32 (36.0) | |
| **LDH** | **(n = 89)** | |
| > ULN | 22 (24.7) | |
| ≤ ULN | 67 (75.3) | |
| **CsCa** | **(n = 90)** | |
| > 2.75 mmol/L | 16 (17.8) | |
| ≤ 2.75 mmol/L | 74 (82.2) | |
| **CrCl** | **(n = 90)** | |
| > 60 ml/min | 40 (44.4) | |
| 31-60 ml/min | 20 (22.2) | |
| 21-30 ml/min | 6 (6.7) | |
| < 20 ml/min | 24 (26.7) | |
| **IHD** | **(n = 90)** | |
| Yes | 9 (10.0) | |
| No | 81 (90.0) | |
| **Hemoglobin** | **(n = 90)** | |
| < 100 g/L | 58 (64.4) | |
| ≥ 100 g/L | 32 (35.6) | |
| **Neutrophil** | **(n = 90)** | |
| < 1×10^9^/L | 5 (5.6) | |
| ≥ 1×10^9^/L | 85 (94.4) | |
| **Platelet** | **(n = 90)** | |
| < 75×10^9^/L | 9 (10.0) | |
| 75-99×10^9^/L | 7 (7.8) | |
| ≥ 100×10^9^/L | 74 (82.2) | |
| **Extramedullary disease** | **(n = 90)** | |
| Yes | 29 (32.2) | |
| No | 61 (67.8) | |
| **Bone lesion** | **(n = 89)** | |
| Yes | 56 (62.9) | |
| No | 33 (37.1) | |
| **FISH** |  | |
| del(17p) | 8/84 (9.5) | |
| del(13q) | 32/75 (42.7) | |
| 1q+ | 49/84 (58.3) | |
| t(11;14) | 18/74 (24.3) | |
| t(4;14) | 10/74 (13.5) | |
| t(14;16) | 0/74 (0) | |
| HRCA^a^ | 15/74 (20.3) | |
| SRCA | 59/74 (79.7) | |
| **ECOG PS** | **(n = 90)** | |
| 0-1 | 39 (43.3) | |
| 2 | 17 (18.9) | |
| 3 | 29 (32.2) | |
| 4 | 5 (5.6) | |
| **Karnofsky performance** |  | |
| > 70 | 46 (51.1) | |
| ≤ 70 | 44 (48.9) | |

Abbreviations: ISS, International Staging System; R-ISS, revised ISS; BMPC, bone marrow plasma cells; β2-MG, β2-microglobulin; LDH, lactate dehydrogenase; ULN, the upper limit of normal; CsCa, serum corrected calcium; CrCl, creatinine clearance; IHD, intermittent hemodialysis; FISH, fluorescence in situ hybridization; 1q+, 1q gain/amplification; HRCA, high risk cytogenetic abnormality; SRCA, standard risk cytogenetic abnormality; ECOG PS, Eastern Cooperative Oncology Group Performance Status.

^a^Including del(17p), t(4;14), and t(14;16) according to the R-ISS.

**Table S2.** **Baseline frailty scores (n = 90)**

| **Frailty score** | | **N (%)** |
| --- | --- | --- |
| **IMWG-FI** | |  |
| Fit | 33 (36.7) | |
| Intermediate fit | 16 (17.8) | |
| Frail | 41 (45.6) | |
| Frail based on age alone | 1 (1.1) | |
| Frail based on ADL/IADL | 22 (24.4) | |
| Frail based on other parameters | 18 (20.0) | |
| **IMWG-FI score** |  | |
| 0 | 33 (36.7) | |
| 1 | 16 (17.8) | |
| 2 | 27 (30.0) | |
| 3 | 11 (12.2) | |
| 4 | 3 (3.3) | |
| **CCI** |  | |
| 0 | 47 (52.2) | |
| 1 | 25 (27.8) | |
| 2 | 12 (13.3) | |
| 3 | 6 (6.7) | |
| **ADL** |  | |
| > 4 | 51 (56.7) | |
| ≤ 4 | 39 (43.3) | |
| **IADL** |  | |
| > 5 | 49 (54.4) | |
| ≤ 5 | 41 (45.6) | |

Abbreviations: IMWG-FI, International Myeloma Working Group Frailty Index; ADL, Activity of Daily Living; IADL, Instrumental Activity of Daily Living; CCI, Charlson Comorbidity Index.

**Table S3. Baseline characteristics according to frailty categorization**

| **Characteristics** | | **Fit, N (%)** | | **Intermediate fit, N (%)** | | **Frail, N (%)** | |
| --- | --- | --- | --- | --- | --- | --- | --- |
| **Age, years** | | **(n = 33)** | **(n = 16)** | | | | **(n = 41)** |
| Median (range) | 68 (65-75) | | | | 68 (65-77) | | 72 (65-87) |
| 65-70 | 26 (78.8) | | | | 10 (62.5) | | 17 (41.5) |
| 71-75 | 7 (21.2) | | | | 5 (31.3) | | 13 (31.7) |
| 76-80 | 0 (0) | | | | 1 (6.3) | | 7 (17.1) |
| > 80 | 0 (0) | | | | 0 (0) | | 4 (9.8) |
| **Sex** | **(n = 33)** | | | | **(n = 16)** | | **(n = 41)** |
| Female | 15 (45.5) | | | | 9 (56.3) | | 20 (48.8) |
| Male | 18 (54.5) | | | | 7 (44.8) | | 21 (51.2) |
| **M protein** | **(n = 33)** | | | | **(n = 16)** | | **(n = 41)** |
| IgG | 15 (45.5) | | | | 6 (37.5) | | 17 (41.5) |
| IgA | 7 (21.2) | | | | 3 (18.8) | | 9 (22.0) |
| IgD | 3 (9.1) | | | | 0 (0) | | 3 (7.3) |
| Light chain | 7 (21.2) | | | | 6 (37.5) | | 10 (24.4) |
| Non/oligosecretory | 1 (3.0) | | | | 1 (6.3) | | 2 (4.9) |
| **ISS** | **(n = 33)** | | | | **(n = 16)** | | **(n = 40)** |
| I | 3 (9.1) | | | | 3 (18.8) | | 1 (2.5) |
| II | 15 (45.5) | | | | 3 (18.8) | | 7 (17.5) |
| III | 15 (45.5) | | | | 10 (62.5) | | 32 (80.0) |
| **R-ISS** | **(n = 32)** | | | | **(n = 14)** | | **(n = 37)** |
| I | 1 (3.1) | | | | 2 (14.3) | | 1 (2.7) |
| II | 23 (71.9) | | | | 10 (71.4) | | 22 (59.5) |
| III | 8 (25.0) | | | | 2 (14.3) | | 14 (37.8) |
| **BMPC** | **(n = 33)** | | | | **(n = 16)** | | **(n = 41)** |
| ≥ 30% | 16 (48.5) | | | | 9 (56.3) | | 28 (68.3) |
| < 30% | 17 (51.5) | | | | 7 (43.8) | | 1 (31.7) |
| **β2-MG** | **(n = 33)** | | | | **(n = 16)** | | **(n = 40)** |
| ≥ 5.5 mg/L | 15 (45.5) | | | | 10 (62.5) | | 32 (80.0) |
| < 5.5 mg/L | 18 (54.5) | | | | 6 (37.5) | | 8 (20.0) |
| **LDH** | **(n = 33)** | | | | **(n = 16)** | | **(n = 40)** |
| > ULN | 8 (24.2) | | | | 3 (18.8) | | 11 (27.5) |
| ≤ ULN | 25 (75.8) | | | | 13 (81.3) | | 29 (72.5) |
| **CsCa** | **(n = 33)** | | | | **(n = 16)** | | **(n = 41)** |
| > 2.75 mmol/L | 2 (6.1) | | | | 2 (12.5) | | 12 (29.3) |
| ≤ 2.75 mmol/L | 31 (93.9) | | | | 14 (87.5) | | 29 (70.7) |
| **CrCl** | **(n = 33)** | | | | **(n = 16)** | | **(n = 41)** |
| > 60 ml/min | 20 (60.6) | | | | 7 (43.8) | | 13 (31.7) |
| 31-60 ml/min | 6 (18.2) | | | | 6 (37.5) | | 8 (19.5) |
| 21-30 ml/min | 1 (3.0) | | | | 1 (6.3) | | 4 (9.8) |
| < 20 ml/min | 6 (18.2) | | | | 2 (12.5) | | 16 (39.0) |
| **IHD** | **(n = 33)** | | | | **(n = 16)** | | **(n = 41)** |
| Yes | 3 (9.1) | | | | 1 (6.3) | | 5 (12.2) |
| No | 30 (90.9) | | | | 15 (93.8) | | 36 (87.8) |
| **Hemoglobin** | **(n = 33)** | | | | **(n = 16)** | | **(n = 41)** |
| < 100 g/L | 16 (48.5) | | | | 10 (62.5) | | 32 (78.0) |
| ≥ 100 g/L | 17 (51.5) | | | | 6 (37.5) | | 9 (22.0) |
| **Neutrophil** | **(n = 33)** | | | | **(n = 16)** | | **(n = 41)** |
| < 1×10^9^/L | 3 (9.1) | | | | 2 (12.5) | | 0 (0) |
| ≥ 1×10^9^/L | 30 (90.9) | | | | 14 (87.5) | | 41 (100) |
| **Platelet** | **(n = 33)** | | | | **(n = 16)** | | **(n = 41)** |
| < 75×10^9^/L | 4 (12.1) | | | | 1 (6.3) | | 4 (9.8) |
| 75-99×10^9^/L | 2 (6.1) | | | | 2 (12.5) | | 3 (7.3) |
| ≥ 100×10^9^/L | 27 (81.8) | | | | 13 (81.3) | | 34 (82.9) |
| **Extramedullary disease** | **(n = 33)** | | | | **(n = 16)** | | **(n = 41)** |
| Yes | 10 (30.3) | | | | 5 (31.3) | | 14 (34.1) |
| No | 23 (69.7) | | | | 11 (68.8) | | 27 (65.9) |
| **Bone lesion** | **(n = 33)** | | | | **(n = 16)** | | **(n = 40)** |
| Yes | 18 (54.5) | | | | 7 (43.8) | | 31 (77.5) |
| No | 15 (45.5) | | | | 9 (56.3) | | 9 (22.5) |
| **FISH** |  | | | |  | |  |
| del(17p) | 5/32 (15.6) | | | | 0/13 (0) | | 3/39 (7.7) |
| del(13q) | 12/28 (42.8) | | | | 7/12 (58.3) | | 13/35 (37.1) |
| 1q+ | 16/32 (50.0) | | | | 8/13 (61.5) | | 25/39 (64.1) |
| t(11;14) | 8/30 (26.7) | | | | 2/11 (18.2) | | 8/33 (24.2) |
| t(4;14) | 4/30 (13.3) | | | | 0/11 (0) | | 6/33 (18.2) |
| t(14;16) | 0/30 (0) | | | | 0/11 (0) | | 0/33 (0) |
| HRCA^a^ | 7/30 (23.3) | | | | 0/11 (0) | | 8/33 (24.2) |
| SRCA | 23/30 (76.7) | | | | 11/11 (100) | | 25/33 (75.8) |
| **ECOG PS** | **(n = 33)** | | | | **(n = 16)** | | **(n = 41)** |
| < 2 | 25 (75.8) | | | | 12 (75.0) | | 2 (4.9) |
| ≥ 2 | 8 (24.2) | | | | 4 (25.0) | | 39 (95.1) |
| **Karnofsky performance** | **(n = 33)** | | | | **(n = 16)** | | **(n = 41)** |
| > 70 | 30 (90.9) | | | | 11 (68.8) | | 5 (12.2) |
| ≤ 70 | 3 (9.1) | | | | 5 (31.3) | | 36 (87.8) |

Abbreviations: M protein, monoclonal protein type; ISS, International Staging System; R-ISS, revised ISS; BMPC, bone marrow plasma cells; β2-MG, β2-microglobulin; LDH, lactate dehydrogenase; ULN, the upper limit of normal; CsCa, serum corrected calcium; CrCl, creatinine clearance; IHD, intermittent hemodialysis; FISH, fluorescence in situ hybridization; 1q+, 1q gain/amplification; HRCA, high risk cytogenetic abnormality; SRCA, standard risk cytogenetic abnormality; ECOG PS, Eastern Cooperative Oncology Group Performance Status.

^a^Including del(17p), t(4;14), and t(14;16) according to the R-ISS.

**Table S4. Treatment discontinuation according to induction cycles**

| **Induction** | **All, N (%)** | **Fit, N (%)** | **Intermediate fit, N (%)** | **Frail, N (%)** |
| --- | --- | --- | --- | --- |
| **Cycle 1** | **(n = 88)** | **(n = 32)** | **(n = 16)** | **(n = 40)** |
| Adverse events | 10 (11.4) | 2 (6.3) | 1 (6.3) | 7 (17.5) |
| Deteriorating condition | 2 (2.3) | − | − | 2 (5.0) |
| Noncompliance | 1 (1.1) | − | − | 1 (2.5) |
| **Cycle 2** | **(n = 75)** | **(n = 30)** | **(n = 15)** | **(n = 30)** |
| Adverse events | 4 (5.3) | − | − | 4 (13.3) |
| **Cycle 3** | **(n = 70)** | **(n = 30)** | **(n = 15)** | **(n = 25)** |
| COVID-19 | 2 (2.9) | 1 (3.3) | − | 1 (4.0) |
| Deteriorating condition | 2 (2.9) | − | 1 (6.7) | 1 (4.0) |
| Progression | 1 (1.4) | − | 1 (6.7) | − |
| **Cycle 4** | **(n = 62)** | **(n = 27)** | **(n = 12)** | **(n = 23)** |
| Adverse events | 3 (4.8) | 2 (7.4) | − | 1 (4.3) |
| Noncompliance | 1 (1.6) | − | − | 1 (4.3) |
| **Cycle 5** | **(n = 56)** | **(n = 24)** | **(n = 12)** | **(n = 20)** |
| Progression | 1 (1.8) | − | − | 1 (5.0) |
| Noncompliance | 2 (3.6) | 1 (4.2) | − | 1 (5.0) |
| **Cycle 6** | **(n = 49)** | **(n = 21)** | **(n = 11)** | **(n = 17)** |
| Noncompliance | 1 (2.0) | − | 1 (9.1) | − |
| **Cycle 7** | **(n = 43)** | **(n = 20)** | **(n = 8)** | **(n = 15)** |
| Adverse events | 1 (2.3) | 1 (5.0) | − | − |
| COVID-19 | 1 (2.3) | 1 (5.0) | − | − |
| **Cycle 8** | **(n = 38)** | **(n = 16)** | **(n = 8)** | **(n = 14)** |
| Progression | 1 (2.6) | 1 (6.3) | − | − |
| Noncompliance | 1 (2.6) | 1 (6.3) | − | − |

**Table S5. Grade 2-4 adverse events during induction (n = 90)**

|  | | **CTCAE grade** | | | | |
| --- | --- | --- | --- | --- | --- | --- |
| **Adverse event** | | **2, N (%)** | **3, N (%)** | | | **4, N (%)** |
| **Non-hematologic AE, any** | 22 (24.4) | | | 31 (34.4) | 12 (13.3) | |
| Infection | 25 (27.8) | | | 24 (26.7) | 10 (11.1) | |
| Pneumonia | 5 (5.6) | | | 23 (25.6) | 6 (6.7) | |
| Bronchitis | 3 (3.3) | | | − | − | |
| Sepsis | − | | | 1 (1.1) | 4 (4.4) | |
| Urinary tract infection | 5 (5.6) | | | − | − | |
| Cytomegaloviremia | 7 (7.8) | | | − | − | |
| Epstein-barr viremia | 2 (2.2) | | | − | − | |
| Herpes zoster | 2 (2.2) | | | 2 (2.2) | − | |
| Influenza A | 2 (2.2) | | | − | − | |
| GI | 14 (15.6) | | | 10 (11.1) | − | |
| Diarrhea | 7 (7.8) | | | 8 (8.9) | − | |
| Constipation | 3 (3.3) | | | − | − | |
| Ileus | − | | | 1 (1.1) | − | |
| Emesis | 1 (1.1) | | | − | − | |
| Acute gastroenteritis | 3 (3.3) | | | 1 (1.0) | − | |
| GI hemorrhage | 1 (1.1) | | | − | − | |
| Cardiac | 1 (1.1) | | | 4 (4.4) | 1 (1.1) | |
| Acute heart failure | − | | | 4 (4.4) | 1 (1.1) | |
| Cardiac arrhythmia | 1 (1.1) | | | − | − | |
| CNS | − | | | 1 (1.1) | − | |
| Cerebral infarction | − | | | 1 (1.1) | − | |
| PNP^a^ | 8 (8.9) | | | − | − | |
| Rash | 10 (11.1) | | | − | − | |
| DVT | 17 (18.9) | | | − | − | |
| Others | 4 (4.4) | | | − | 1 (1.1) | |
| Dizziness | 1 (1.1) | | | − | − | |
| Anxiety | 3 (3.3) | | | − | − | |
| Hyperkalemia | − | | | − | 1 (1.1) | |
| COVID-19 | 6 (6.7) | | | 5 (5.6) | − | |
| **Hematologic AE,** **any** | 17 (18.9) | | | 29 (32.2) | 16 (17.8) | |
| Anemia | 3 (3.3) | | | 10 (11.1) | 1 (1.1) | |
| Neutropenia | 31 (34.4) | | | 21 (23.3) | 4 (4.4) | |
| Thrombocytopenia | 17 (18.9) | | | 18 (20.0)^b^ | 14 (15.6)^b^ | |

Abbreviations: CTCAE, Common Terminology Criteria for Adverse Events; AE, adverse event; GI, gastrointestinal toxicity; CNS, central nervous system; PNP, peripheral neuropathy; DVT, deep vein thrombosis.

^a^Grade 1 PNP was observed in 22 (24.4%) patients.

^b^Including one patient with cerebral hemorrhage secondary to thrombocytopenia.

**Table S6. Grade 2-4 adverse events according to frailty categorization**

| **Adverse event** | **Fit**  **(n = 33)** | | | | **Intermediate fit**  **(n = 16)** | | | | **Frail**  **(n = 41)** | | |
| --- | --- | --- | --- | --- | --- | --- | --- | --- | --- | --- | --- |
|  | **G2, N** | **G3, N** | **G4, N** | **G2, N** | | **G3, N** | **G4, N** | **G2, N** | | **G3, N** | **G4, N** |
| **Non-hematologic AE** |  |  |  |  | |  |  |  | |  |  |
| Any | 12 | 11 | 4 | 2 | | 7 | 1 | 8 | | 13 | 7 |
| Infection | 5 | 7 | 2 | 5 | | 4 | 1 | 15 | | 13 | 7 |
| Pneumonia | − | 6 | 1 | 1 | | 4 | 1 | 4 | | 13 | 4 |
| Bronchitis | − | − | − | 1 | | − | − | 2 | | − | − |
| Sepsis | − | 1 | 1 | − | | − | − | − | | − | 3 |
| Urinary tract infection | 1 | − | − | 1 | | − | − | 3 | | − | − |
| Cytomegaloviremia | 4 | − | − | 1 | | − | − | 2 | | − | − |
| Epstein-barr viremia | − | − | − | 1 | | − | − | 1 | | − | − |
| Herpes zoster | − | 1 | − | − | | 1 | − | 2 | | − | − |
| Influenza A | − | − | − | 1 | | − | − | 1 | | − | − |
| GI | 6 | 5 | − | 4 | | 2 | − | 4 | | 3 | − |
| Diarrhea | 3 | 4 | − | 3 | | 2 | − | 1 | | 2 | − |
| Constipation | 1 | − | − | 1 | | − | − | 1 | | − | − |
| Ileus | − | 1 | − | − | | − | − | − | | − | − |
| Emesis | − | − | − | − | | − | − | 1 | | − | − |
| Acute gastroenteritis | 1 | − | − | − | | − | − | 2 | | 1 | − |
| GI hemorrhage | 1 | − | − | − | | − | − | − | | − | − |
| Cardiac | − | 2 | 1 | − | | − | − | 1 | | 2 | − |
| Acute heart failure | − | 2 | 1 | − | | − | − | − | | 2 | − |
| Cardiac arrhythmia | − | − | − | − | | − | − | 1 | | − | − |
| CNS | − | − | − | − | | − | − | − | | 1 | - |
| Cerebral infarction | − | − | − | − | | − | − | − | | 1 | − |
| PNP^a^ | 5 | − | − | 2 | | − | − | 1 | | − | − |
| Rash | 8 | − | − | 1 | | − | − | 1 | | − | − |
| DVT | 5 | − | − | 3 | | − | − | 9 | | − | − |
| Others | 1 | − | 1 | − | | − | − | 3 | | − | - |
| Dizziness | − | − | − | − | | − | − | 1 | | − | − |
| Anxiety | 1 | − | − | − | | − | − | 2 | | − | − |
| Hyperkalemia | − | − | 1 | − | | − | − | − | | − | − |
| COVID-19 | 4 | 2 | − | 1 | | 1 | − | 1 | | 2 | − |
| **Hematologic AE** |  |  |  |  | |  |  |  | |  |  |
| Any | 8 | 14 | 3 | 1 | | 6 | 5 | 8 | | 9 | 8 |
| Anemia | − | 5 | − | 1 | | 2 | − | 2 | | 3 | 1 |
| Neutropenia | 12 | 9 | − | 6 | | 4 | 3 | 13 | | 8 | 1 |
| Thrombocytopenia | 6 | 7 | 3 | 4 | | 5 | 3 | 7 | | 6^b^ | 8 |

Abbreviations: CTCAE: Common Terminology Criteria for Adverse Events; AE: adverse event; GI: gastrointestinal toxicity; CNS: central nervous system; PNP: peripheral neuropathy; DVT: deep vein thrombosis.

^a^Grade 1 PNP was observed in 10, 5, and 7 patients in the fit, intermediate fit, and frail groups, respectively.

^b^Including 1 patient was combined with cerebral hemorrhage due to thrombocytopenia.

**Figure Legends**

**Fig. S1.** Diagram for the dynamic frailty-tailored therapy (DynaFiT) according to longitudinal changes in the frailty category defined at baseline and before the start of each cycle.

*Daratumumab 16 mg/kg intravenously (cycle 1-2: days 1, 8, 15, and 22; cycle 3-6: days 1 and 15; cycle 7-8: day 1), followed by maintenance with daratumumab (day 1), was recommended for frail patients. The dose and schedule of daratumumab were not adjusted or discontinued when frail patients became fit or intermediate fit.

**Fig. S2. Patient flow through the study**. Flowchart of patients participating in this study, through eight induction cycles (cy 1-8) and timing and reason for treatment discontinuation.

^a^One patient discontinued treatment because of cerebral hemorrhage secondary to thrombocytopenia.

PNP-P, peripheral neuropathy grade 2 with pain.

**Fig. S1**

**Fig. S2**
